# Conflicting Interests and New Frontiers: A Role for Virtue Ethics in Cutting Edge Brain Research with Humans

**DOI:** 10.1007/s12152-025-09618-0

**Published:** 2025-10-21

**Authors:** Ally Peabody Smith, Lilyana Levy, Colleen Hanson, Nader Pouratian, Ashley Feinsinger

**Affiliations:** 1 Department of Population Health, College of Health, Lehigh University, Bethlehem, USA; 2 Department of Neurosurgery, David Geffen School of Medicine, University of California, Los Angeles, USA; 3 Department of Philosophy, University of California, Los Angeles, USA; 4 Department of Neurological Surgery, UT Southwestern Medical Center, Dallas, USA

**Keywords:** Neuroethics, Conflicts of interest, Neuroscience, Research ethics, Human subjects research, Neurosurgery, Virtue ethics

## Abstract

**Background:**

One of the central goals of recent neuroethics research is to understand the ethical implications that rapidly evolving neuroscientific discoveries and technologies may have for research participants, patients, and society. From adequate informed consent and post-trial obligations to impacts on agency and disability justice, neuroethicists have argued that the unique clinical, investigative, and financial context of these advances raises distinct and urgent ethical challenges. While much of the ethics work has been advanced through soliciting the perspectives of patients, participants, and the broader public, comparatively little work has explored the experiences of researchers who lead these studies, what ethical issues they face, and how they navigate them. Compared to other parties, investigators are uniquely situated with respect to both their agency and experience. They are the only stakeholders who experience ethical issues before, during, and after a study, and who interact with industry, universities, federal agencies, funders, IRBs, medical centers, ethicists, patients, participants, and caregivers. They are also in a position to determine, with relative authority, the designs of their studies and the solutions to ethical issues as they arise. Understanding researcher experiences is thus a critical part of recognizing, navigating, and mitigating the ethical issues that arise in cutting edge brain research.

**Methods:**

We conducted a qualitative study with 14 NIH BRAIN Initiative-funded investigators, whose research spans 11 institutions and which involves various kinds of invasive brain research with humans. Interviewees were asked about their experiences conducting research and responding to ethical challenges, navigating academic and commercial institutions, interacting with patients, participants, and other researchers, and engaging with neuroethicists. Interviews were coded and analyzed utilizing an inductive and semantic reflexive thematic analysis.

**Results:**

Analyses of interviews yielded two main themes: (i) the difficulty of navigating complex conflicts of interest and (ii) the need for collaboration, community, and participation in neuroethics deliberation. Researchers describe facing multiple underappreciated structural and interpersonal conflicts of interest (including those from research funding, team structure, data collection and sharing obligations, commercialization, innovation, and the boundaries between research and care), as well as the need for increased engagement with participants, ethicists, and each other.

**Conclusions:**

Drawing on this data, we argue that navigating ethical issues in cutting edge brain research requires a shift from focusing on promoting ethical guidelines to also promoting neuroethical competencies. More specifically, we argue that integrating the philosophical discipline of virtue ethics—which focuses on developing moral sensitivities, practical reasoning skills, and other ethical competencies—could provide better tools for addressing the ethical issues researchers face than regulations and rules alone. We conclude by suggesting two ways that neuroethicists and neuroscientists might move together towards a virtue ethical ideal: (i) promoting targeted neuroethics education and opportunities for participating in collective moral deliberation, and (ii) prioritizing engagement amongst researchers, ethicists, and study participants.

## Introduction

Rapid advances in neurotechnology and neuroscientific inquiry across clinical, scientific, and commercial domains have led to significant discoveries about the brain and novel treatments for brain disorders [1–6]. New assistive technologies have been advanced for individuals with spinal cord injuries and ALS, including brain computer interfaces to control robotic limbs and facilitate communication. Treatments for movement disorders like Parkinson’s disease, essential tremor, and dystonia have been advanced through deep brain stimulation, and similar trajectories are emerging for obsessive compulsive disorder and depression. Clinical researchers have advanced our understanding of the fundamental neural processes that underwrite memory, language, and cognition, facilitating new applications for existing technologies as well as expanding possibilities for new ones [7–15].

Many of these advances have been made possible through invasive research with human participants. Patients who undergo invasive interventions for neurological or psychiatric disorders offer researchers the unique opportunity to directly study and modulate the human brain using implanted wires and devices. For example, a patient undergoing a brain surgery to implant a deep brain stimulation device for the treatment of Parkinson’s disease might consent to participate in research that takes advantage of the implanted technologies and the unique surgical context of their operation. Similarly, patients in the epilepsy monitoring unit might participate in a study which makes use of electrodes implanted in their brains to monitor seizure activity, or might consent to the placement of modified electrodes which enable specific research studies. Individuals who have adopted early versions of brain computer interfaces, such as the Orion visual cortical prosthesis system or Neuralink’s N1 implant, offer researchers additional opportunities to study the brain through these implanted devices. Importantly, much of this research would not otherwise be permissible, since the risks of invasive techniques are normally too great to license research or technology development alone.

Neuroethicists have raised a host of ethical issues related to this research. The urgency to address them grows alongside rising investments in neurotechnology from public, private, and philanthropic funding agencies [16–25]. Neuroethicists have studied whether this research poses a threat to voluntary consent given that many principal investigators are also the treating surgeons [26, 27], and whether participants, perhaps more focused on the risks and outcomes of their brain surgery or diagnostic monitoring, adequately understand the separation of research and care [28–30]. They have questioned what should happen to device trial participants after their trials are over, [31–35] appropriate data sharing practices, [36] how participating in a novel device trial might impact a person’s identity, [37–39] and what the effects of emerging neurotechnologies are on intimate aspects of the human experience, including one’s sense of self, agency, and relation to the world [40].

Notably, a significant portion of this work has been carried out through soliciting the experiences and perspectives of patients, research participants, and the public, which have in turn been leveraged to lobby for better research practices and the adoption of new guidelines and regulations [41, 42]. The present study is motivated by the fact that so far, less attention has been paid to understanding the perspectives of investigators regarding their experiences designing and carrying out invasive studies with humans. While some work has documented investigator perspectives on specific ethical issues like recruitment and consent [43], engagement with device users [44, 45], and the permissibility of certain technologies [46, 47], there exist few *broad inquiries into the experiences of investigators conducting invasive brain research*. Rather than assuming investigator perspectives align with those of ethicists, patients, participants, and the public, this study begins from the position that investigators are a unique group whose perspectives should shape any attempt to improve the ethics of invasive brain research.

Investigator perspectives are important for neuroethical progress for at least two reasons. First, investigators are uniquely situated in terms of their ***agency***. Compared to other stakeholders, they possess significant control over the details of their studies, including how to address ethical issues that arise in designing, carrying out, and managing the post-trial impact of their studies. Second, investigators are uniquely situated in terms of their ***experience***. They interact with multiple patients and participants, across multiple studies, and sometimes across many institutions. They are the only group that simultaneously interacts with industry, federal agencies, funders, IRBs, medical centers, ethicists, patients, participants, and caregivers throughout the duration of a study. From this vantage point, they may experience new dimensions of familiar issues or be able to characterize new issues altogether.

For these reasons, we conducted an inquiry into the perspectives and experiences of 14 NIH BRAIN Initiative principal investigators conducting invasive brain research with humans. Interviewees were asked a broad range of questions about their perspectives, including what it’s like conducting their research, what ethical issues they identify, how they navigate ethical challenges, how they traverse research, academic, and commercial institutions, and how they engage with patients, participants, and neuroethicists. The study’s primary aim was to explore how investigators experience ethical issues in their studies and how these experiences might direct neuroethics to different or new approaches.

Below, we present the resulting narratives, which describe multiple ethical pressures that research teams continue to face despite emerging guidelines and regulations. We highlight two major themes: (i) the difficulty of navigating complex conflicts of interest and (ii) the need for collaboration, community, and participation in neuroethics practice. Drawing upon these interviews, we argue that current approaches to neuroethics are insufficient, and that embracing core features of virtue ethics would better help researchers navigate the issues they describe.

## Methods

### Participants

We conducted semi-structured interviews via Zoom with NIH BRAIN Initiative-funded principal investigators (n = 14) across 11 institutions who were involved in various kinds of invasive brain research with humans. The research conducted by interviewees included intraoperative research, research in the epilepsy monitoring unit, and brain computer interface research, sometimes carried out in conjunction with non-invasive techniques including fMRI and transcranial magnetic stimulation. Interviewees also occupied a variety of professional roles, including clinician-researcher, non-clinician researcher, engineer, neuroscientist, and neurosurgeon. Expertise and research focus also varied, including concentrations on central nervous system disorders (such as Parkinson’s disease, epilepsy, and tetraplegia), language acquisition, memory formation, sensory processing, and topics in auditory cognitive neuroscience. Interviewees were recruited and consented on a rolling basis via email, using a modified purposeful snowballing sampling [48]. Members of the study team reached out to existing contacts through the Research Opportunities with Humans Consortium of the NIH and requested referrals to additional potential interviewees. The study was approved by the Institutional Review Boards at University of California, Los Angeles and University of Texas, Southwestern. Interviews have been anonymized and will be referred to as principal investigator (P) followed by a number between 1–14, i.e. P4, P11.

### Interview Constructs

Interviewees were asked a series of open-ended questions beginning with the nature of their research and general experiences, which then directed the interviewer to a series of ethics-oriented questions pertinent to the interviewee’s own research. Topics included: experiences and challenges related to risk, recruitment, and consent; views about the ethical significance of current neuroscience research with humans, including basic and non-therapeutic studies; views about participant motivations, experiences, and values; experiences interacting and communicating with participants and with other researchers; views about the value of participant and stakeholder engagement; and the role of investigators and ethicists in neuroethics research and practice. All interviews were conducted by APS in November and December of 2022.

### Analysis

Recorded Zoom interviews lasted between 40 and 70 min and were transcribed using Rev Transcription Services. Transcripts were read by all coding team members (APS, LL, CH, AF) with the objective of identifying codes representative of the content and topics covered in the interviews. Team members then met to iteratively revise the codes to find consensus. Four interviews were test coded by two team members per interview, after which all team members completed a final revision of the codebook for efficacy and completeness. Interviews were then coded with the finalized codebook, again using two coders per interview. Inconsistencies in coding were discussed and revised via one-on-one coding resolution meetings.

Using inductive and semantic reflexive thematic analysis [49–52], team members identified overarching themes that captured the conceptual content of the interviews across codes. Information was then progressively abstracted by team members via the identification of high-impact representative quotes from each theme. Reflexive thematic analysis admits the influence of investigator perspectives and training, which, in the case of this study, included philosophy, bioethics, and disability studies. As such, analysis was shaped by conceptual and ethical concerns.

## Results

Thematic analysis yielded two overarching themes of ethical interest. The first was ***the difficulty of navigating complex conflicts of interest***, despite increased attention to the ethics of their research. Interviewees described tensions arising from multiple considerations, which in some cases made aspects of research and patient care difficult or left them unsure of how to proceed. Below, we divide these conflicts of interest into two subthemes, which correspond to the different sources of pressure described by investigators: conflicts arising from structural factors (Table 1) and conflicts arising from the boundaries between research and care (Table 2).

The second theme was ***the need for collaboration, community, and participation in neuroethics practice*** (Table 3). Researchers described past experiences, concerns, and hopes related to the role of ethicists in cutting edge brain research, the value of engagement with ethicists, participants, and other stakeholders, and the need for researchers to collaborate and participate in ethical conversations.

### Conflicts Arising from Structural Factors

When asked general questions about their experiences conducting invasive research with humans, as well as when describing challenging situations they experienced, many investigators discussed conflicting pressures emerging from how academia, grant funded research, and clinical care are incentivize and organized, making demands on how they conduct science, relate to participants, and engage with biotechnology companies. While many of these pressures were discussed in overlapping ways, we distill four main areas of structural pressures below, and present representative quotes in Table 1, *Conflicts arising from structural factors.*

**Table 1:** Conflicts arising from structural factors

Grant funding	We have to get grants or we lose our jobs… I think there’s certainly a conflict there, in the sense that we need the data in furtherance of our own careers. Obviously, if you are also the person treating the patient, there’s a next level conflict there… Just speaking hypothetically, if you’re a surgeon and you’re submitting a grant, and you need data, and this is your patient, and the patient knows you’re the surgeon, right? There are some things you can’t be blinded to. And so whenever you can’t be blinded to something, I think you need to be aware of it. I don’t think it necessarily disqualifies the task, but certainly something to think about from a process standpoint. (P13)
	Data release is really absolutely critical because no single investigator will ever explore all facets of a given dataset. That that’s never going to happen. So, it would really profit others to release those datasets so that these other facets can be looked at by others if they’re of interest. And that of course is quite a difficult issue because, as an investigator, you’re thinking, well, I just wrote five grants to fund this work, and worked on it for five years, so why should I give away this data? But I think in the broader picture that that’s absolutely the right thing to do. (P3)
Academic research practices	As the BRAIN Initiative grows, people are less and less well-rounded. In other words, if you look at larger programs, it becomes less likely that they have been part of every little step. So, what that means is that they think, and this is actually dangerous, they actually think they can do everything. They spread their wings, they become independent, and they go to smaller sites with less oversight or less institutional knowledge, and they start doing the things that they have learned about but not the things that they have not learned about. And I think the big problem is how they proliferate it across other sites or in the industry… There are sites that do this research, and I’ve visited some of those, [where training is] completely hands-off. And then there are sites where basically the PI [manages the trainee’s] schedule and makes sure that [they are] fully dedicated to the research. This is a problem in some ways because it requires a lot of protected time. But I think that’s the field of tension that we are in with advancing technology, making it more portable to make sure that [trainees] know what to do with it, and having the resources, especially time, to supervise to appropriately deploy them. (P6)
	Nobody checks neurosurgeons’ work. It just doesn’t happen. And that seems weird. It’s like there’s this big move towards transparency and replicability in all sorts of areas of research. And for many, many research approaches, it’s feasible to reproduce the study… Intracranial work you can’t replicate. Even if you have a similar population, there’s not enough incentive to try to replicate others’ findings because people all want to publish *Nature* and *Science* papers and whatever, and so they want to do their cool new thing. So, I feel like a lot of scientific findings coming out of this work also don’t undergo the same scrutiny as a lot of other work. (P10)
Commercial interests and technological innovation	There are a few thousand patients a year undergoing epilepsy surgery. And there are more than a thousand companies and startups right now, and people interested in creating technologies around those data and around those [limited] patients. (P2, quote A)
	There’s a famous neurosurgeon named Dobelle who claimed he could cure people’s blindness… The patients would fly to Portugal and he would do neurosurgery on them there and put in this crazy device, which didn’t actually work. It would give some of them epilepsy because it was stimulating their brain so much. This is a huge ethical quagmire because, of course, he had a company and he had a paper in *Science*. Not to say he didn’t believe in it, just like Musk may believe in Neuralink, but that’s kind of beside the point. There’s a big conflict of interest because if Elon Musk is in charge of the clinical trial, he’s going to pressure patients to participate and overpromise the benefits. And that’s sort of the worst-case I can imagine. (P5)
	… this field is a little cutthroat. It’s like everybody wants to be first. Nobody trusts anyone who talks about the research. (P6)
	There is this enormous pressure, I think, on many groups, many labs to be as avant-garde with technology and as industrious about it as possible. None of those things necessarily coincide with what is in the patient’s interest… I’m not here to advocate for policing or whatever, but there’s an accelerator around doing innovative, disruptive recordings that gets you funded, gets you notoriety, gets you *Nature* and *Science* papers. Where is the brake? The stronger the accelerator the stronger the brake should be, and we’ve not created a brake. (P2, quote B)
Data collection and privacy	I would say the biggest thing is, we want the data, the data are rare, sort of by definition. You have to be at a major medical facility to get the data. And so anytime you’re kind of set up where the scientist does all of this prep work, and then the patient maybe consents to do research, and then you’re set up and you’re ready to go, and then maybe the patient withdraws consent. Of course, we want them to feel free to do that. So, I would say that’s probably the ethical point there where we tell ourselves we’re not supposed to convince patients to participate. (P13)
	But to some level, I think our participants have more of a desire to be active advocates in this field and sharing their knowledge. And so, I feel that that’s their right to decide how they want to engage with the community, and that does limit our ability to de-identify them to some degree, but that’s their decision… Because we’re working with such small numbers of participants, and a lot of times, their identity becomes known because of media coverage of this study, and it is their decision to participate in that coverage or not. But most of the time they do because their motivation for doing this study is really to help advance these devices…I think the actual risks of being able to identify that data are fairly low. We’re collecting data from 200 channels in motor cortex. It’s such a small population of neurons compared to what’s typically going on there, and it’s from a very local area during very specific tasks. And so, I think that it’s unlikely that there is very sensitive information in that data, but it’s also something that we don’t necessarily know how to describe the potential risk of what could happen if that were released. (P7)

#### Grant Funding

Investigators described how grant funding mechanisms interacted with many other features of their work, creating pressures beyond simply needing to secure grants. These include pressures on career objectives and patient care, and but also on data collection, data sharing, and participant enrollment. Researchers need data to secure grants, and once they get them, they need data to carry out the project. This puts pressure on meeting enrollment numbers but also on getting as much data as possible from those who do enroll. Further, the effort required to get grants and collect rare data interact with pressures to innovate, share data, and publish (P3).

#### Academic Research

Many investigators work at academic hospitals or research centers in which they supervise trainees in neuroscience. To that end, several interviewees mentioned norms within academic neuroscience—including overspecialization, the lack of standardization in training across various practices, the structure of labs, the hierarchies within them (P6), the lack of oversight and transparency (P10), and a lack of sufficient protected time—all as negatively influencing research practices. These factors, which are far more nuanced than the general pressure of producing scholarship, created stress on what researchers thought would be best, either for the science or the participants and patients involved.

#### Commercial Interests and Technological Innovation

Investigators described conflicts emerging from commercial interests in the neurotechnology industry, especially given the scarce opportunities to conduct research and collect high quality data. Notably, these pressures went beyond concerns about patient care when pushed to innovate quickly or design marketable devices, to include pressures created by hype surrounding device development and excitement fueled by public figures (P5). Investigators also discussed the impact these pressures had on creating a research culture of mistrust among researchers, a lack of transparency within the field (P6), and the need for “a brake” to check unmitigated pressures innovate (P2).

#### Data Collection and Privacy

These pressures were multifaceted, including the challenges of balancing the rarity of research opportunities, the ethical obligations of data sharing, and the standards of identity protection. The impact of these pressures reached into other ethical areas, including worries about how the rarity of data collection opportunities may incentivize investigators to inadvertently pressure participants to consent (P13). Concerns about privacy extended beyond the impact of data sharing, to include worries about media coverage and the difficulty of protecting identities in high-profile research (P7). Notably, discussions of data pressures were mentioned across many other topics of discussion, emphasizing the impact of these pressures across the study timeline.

### Conflicts Arising from the Boundaries Between Research and Care

Certain features of the interpersonal relationships between researchers, clinicians, and patients shaped the pressures interviewees felt when conducting their research. Interestingly, investigators expressed heterogenous views regarding the extent to which their relationships with participants mitigated or exacerbated these pressures. These tensions were most robust during discussions of blurred boundaries between research and care, and the dual role of being a clinician-investigator. These narratives were often accompanied with practices used mitigate the pressures of overlapping research and care. Table 2, *Conflicts of interest in the boundaries between research and care*, offers representative quotes discussing the challenges arising from overlapping research and care and example practices and strategies researchers have used to mitigate these pressures.

**Table 2:** Conflicts arising from boundaries between research and care

Blurred boundaries between research and care	This is the litmus test of, “Well, how divorced can research and clinical really be?” If Dr. X is putting electrodes in your brain and he asks you to do something that will help us out if you watch these videos or do this task, then of course you’re going to be likely to agree. But even if it’s not him, if it’s a research assistant, well, you know that research assistant works for Dr. X and so it’s very hard. At what point is it far enough removed? It’s always going to have something to do with your clinical care because you’re only being approached because you’re having electrodes in your brain. (P5)
	I mean, overall, I think that [dual role] relationship is good. It’s very positive because I think it means the physician involved is really trying to push things forward and also develop new things rather than just going with the standard. So, I think that, overall, that’s a good thing because they really have skin in the game in more than one way that way, which is good and it will also really target research where it’s really needed because the physician sees what’s happening. Obviously, it creates ethical issues that one has to be careful, about all the kinds that we have discussed. But in general, I think that’s a good thing and that’s something that NIH very much wants. They want physician investigators to play both roles. They really do. (P3)
	Some people say the surgeon or the clinician should never be part of that consent discussion. I’ve always pushed back against that pretty strongly. I agree that I shouldn’t necessarily be the only person, but I think it’s really impractical to separate it… the patients, they don’t separate it… to them it’s all one thing. And so [potential participants might] start asking the research coordinator about the procedure and the surgery and the recovery and all this sort of stuff. And that person, obviously they can’t respond to that. If they decline to respond, then the patient now all of a sudden feel like there’s no coordination and the team doesn’t know what’s happening… So, there’s some risk of relationship deteriorating, which is not what you want before brain surgery. (P1)
	That’s probably the ethical point, where we tell ourselves we’re not supposed to convince patients to participate… having that line between what is giving them all the information versus what is convincing them. I would say that’s the tricky area. How do you convey that we think the research is valuable? We wouldn’t have a grant from the NIH to do it if we didn’t think it was valuable. But at the same time, your participation is fully voluntary…Again, how do we insulate people from those conflicts of interests, because you can’t eliminate them. If you’re at an academic medical center, physician scientists are a thing. They’re very valuable. (P13)
	I think it really just requires excruciating attention to detail in making absolutely sure that you are presenting the risk–benefit ratio to the participant in as unambiguous a way as you possibly can. And consciously taking your therapeutic relationship out of it. Because you will, by the nature of your treating relationship with the patient, skew the trust ratio in a positive way. And the way I think of it is, I’m going to eliminate that skew as much as I possibly can by going way in the opposite direction. (P12)
	We all have conflicts of interest in various degrees, and it’s important that individuals as well as scientific fields are vigilant about figuring out where to draw the lines, and not assume everything is fine. This is why human-based neuroscience needs to have teams, and why what NIH is doing is great. The research it’s supporting is pushing harder and harder to include clinicians, researchers, neuroethicists, and external advisors. I don’t know whether one person who holds two positions may or may not cross boundaries. It depends on the circumstances…There are advantages to having individuals being able to see the whole project. There are advantages of keeping a division of labor, but I think you always want a thoughtful combination of both. (P11)
Efforts to navigate blurred boundaries	It’s really important that they see me, number one… as their doctor… and that’s how I see myself primarily. So I will, of course, always a hundred percent of the time, sacrifice any part of the research if there’s any question about their well-being. So if they’re just not doing well and they can’t do something, obviously I’m not going to push them to do it. Or if there’s a choice to be made on my end about doing something that might provide more research but might slightly change or increase the risk, I would never do that. (P1)
	When the patients are here, I pretty much try to restrict my role to just being the clinician, try to take care of them, and I let my lab people and the research people talk to the patient about the tasks and the testing and stuff like that… I just don’t want the patient to feel like their research is tied to their clinical care. (P9)
	I may have [care] entirely separate from any research that they may be involved in, but also to make it very clear to the patient that there’s no connection, that they do not need to participate in this to feel that they need to get good clinical care. (P14)
	When the surgeon or the physician talks to the patient, it’s only about their medical care. It helps put that in a box. Then the research chief speaks about research… Sometimes a challenge is to make the patients understand what was research and what was clinical. (P8)
	We often have a clinician involved who knows the patient’s, let’s say seizure for example, seizure behavioral symptoms and how we can best protect the patient from harm if an adverse event were to happen. So that’s definitely a benefit with clinician involvement. (P4)
	We [as researchers] are, of course, not qualified or allowed to make comments on medical questions, which patients often will address to us and we have to be very careful to say, “I will pass it on but I can’t comment on this”… We have found that it’s sometimes the case that the patients feel more comfortable telling us certain things than the clinical care team because they spend many more hours with us. (P3)

#### Blurred Boundaries Between Research and Care

Investigators described multifaceted views about navigating relationships with patients and participants, with a focus on the overlap of research and care. Many investigators noted that the dual roles of being a clinician and a researcher created ethical ambiguity surrounding recruitment and consent, especially when the investigator was involved (P1, P13, P12), including worries about patients feeling pressured to enroll. But narratives went beyond these concerns, to include reflections on whether fully separating research from care was possible (P6), and that despite their efforts, patients ultimately see them together (P1). Others discussed how this overlap is challenging but valuable for the research itself (P3), noting the utility of clinician-investigators’ “birds-eye-view” (P11). In a similar vein, narratives described the importance of trust with participants as positive and necessary, while simultaneously expressing that trust could be taken advantage of, or “skewed” in ways that might affect interpretations of risks and benefits (P12).

#### Efforts to Navigate Blurred Boundaries

Narratives suggest that researchers are also attempting to navigate these boundaries and dual roles in a variety of ways. Interestingly, despite the aforementioned reflections on the positive impact of dual role clinician-investigators, most practical solutions included attempts at separation and prioritization (e.g., prioritizing their role as clinician, separating themselves from research activities whenever possible, assigning specific and separate roles to members of the research team, and having the recruiting individual be a separate individual from the clinician-researcher). In sum, discussions of boundaries and overlapping roles seemed to be a significant source of ethical difficulty, perhaps made more challenging to navigate by the structural pressures of data collection, innovation, grant funding, and academic training.

### The Need for Collaboration, Community, and Participation in Neuroethics Practice

Investigators were also asked how they viewed their own role, as well as the role of neuroethicists and research participants, in making progress on the ethical issues they described. Table 3, *The need for collaboration, community, and participation in neuroethics practice,* shows representative quotes describing experiences with and perspectives on engagement with other investigators, ethicists, and participants.

**Table 3:** The need for collaboration, community, and participation in neuroethics practice

Engaging investigators	What is missing in this field is ethical conversation, and that’s maybe because this field is a little cutthroat. It’s like everybody wants to be first. Nobody trusts anyone who talks about the research. A little bit missing are networks of self-support…and PIs that talk about these issues. (P6)
	No one wants to really take some of these matters head on. It’s like it’s easier to sidestep these matters (and not have anybody calibrating the degrees of divergence from what you might be doing for purely clinical care), than it is to do that [discuss ethical issues]. (P2)
Engaging ethicists	We’ve actually had a lot of discussions about [ethical issues] with our bioethics group here, not for anything formal or official, just because I think that these are issues that we should be talking about… I think philosophers are important. I think physicians are important. I think probably all stakeholders in society should at some extent be playing some sort of role in this discussion. (P9)
	Science progresses when it’s free, but science gets stupid when it’s unfettered. And so there’s some balance. I do think that there’s something special about human neuroscience that requires more careful consideration of these issues than [other research]…I like the idea that now the funding that is driving a lot of this is requiring input from neuroethicists. That’s not quite a regulation, but it’s more than just letting things free run. (P11)
	I’ve learned so much about NIH’s view of incorporating neuroethics and clinical and research considerations and what combination of that constitutes acceptable and unacceptable. I’ve been impressed with the thoughtfulness of the review process about those issues and really considering not just, that’s a cool idea, but is this the right time to be doing that in humans or should you do that in rodents first to just get a better idea of the fundamental question you’re asking and then maybe sharpen it the hypotheses to test in humans? This issue of wearing both the clinical and the research hats comes up in those discussions also. That’s why I said, to me there’s not an a priori priority answer [to these ethical issues], but it depends on what are the checks and balances in place for dealing with the decision making. And in some cases it’s explained appropriately, and in some cases it hasn’t been. And I’m glad that there’s a decision process that allows us to identify those distinctions. (P11)
	…maybe bring people like you [ethicists?] in and help PI’s in addressing the issues rather than basically writing new regulations and say, “Okay, here’s the regulations. Read it and do it.” Because I think a lot of what happens administratively in research is like that. It’s basically, “Here is this thing, do it,” instead of, “Dr. so-and so, we have this issue,” explaining the context…“Do this, do that,” instead of, “Oh, we need to do this because of this and this.” (P6)
	[It helps to] engage with ethicists and people who can help us figure this out from a standpoint of not just gut feelings, but of actual learned study… Not just, what are the philosophical underpinnings here, but what are the policy things? Pragmatic suggestions are helpful for us more concrete guys like neurosurgeons. (P12)
	I think the problem tends to become very quickly that it’s your opinion. This is my opinion and this is my body of work, and all of my body of work depends on this modality and this device. If I don’t do this, then I can’t deliver on my promise. I don’t know exactly where those boundaries are. I’m glad that there are people with sophisticated skill sets like yourself, to evaluate those issues and to give us concrete recommendations hopefully at the end. (P2)
	I’ve had some interactions with [ethicists] and they ask me, basically, they say, “Well, are there actually research questions for me in this as an ethicist or do you just need help in doing applied things?” But they want to do research, of course. What actual ethical research could they actually be doing? They have been asking me that, rather than helping me in being ethical. That’s a totally different thing. (P3)
Engaging with participants	They [participants] went through the actual experience of participating. So they are both familiar with pathology, but also with the treatment itself… [Engagement] would provide another view of the situation, that might enrich the discussion. (P8)
	I think number one is to be informed about what [participant] concerns are with related studies. I think it’s great that other teams are starting to engage with research participants because I can only share my perception of what they think, and they may communicate that differently with other people. And so, I think just being able to hear that, not only from our participants but from others, is very valuable. (P7)
	The current vision, which is not clear if it will happen or not, would be to have them participate in some meetings. To hear a little bit more about their experience, but also to give some feedback about potential task designs or… Basically, have the patient perspective to some of the plans we have. (P8)

#### Engagement Amongst Investigators

Regarding their own involvement in ethical discourse, investigators described a lack of conversation around ethical issues within and between research teams, missing networks of support for addressing ethical challenges, and the fact that researchers can “side-step” these issues instead of collaborating to solve them. These comments mirrored discussions of the impact of data collection and grant funding pressures, which described a culture discouraging of trust, sharing, and collaboration.

#### Engagement with Ethicists

Some interviewees acknowledged that progress was being made on ethical issues in their research thanks to new collaborations between investigators and ethicists, as well as efforts to bring ethicists into the grant review process. Additionally, while some described the value of critical discussions and engagement with ethicists in the past, others expressed a need for ethicists to provide concrete solutions to practical problems and expressed frustration with the lack of current efforts to do so. These narratives might be seen to reflect differing views about what neuroethics should be doing: collaborating *with* researchers to solve ethical challenges together or providing solutions *for* researchers that address real world challenges.

#### Engagement with Participants

Investigators also recognized the value of participant experiences given their first-hand knowledge of what it is like to take part in invasive research and the knowledge they could bring to better study design. Interestingly, investigators did not express skepticism about the value of participant input but hoped that ultimately engagement would increase. These narratives taken togethers suggest that researchers would benefit, perhaps in addressing the conflicting pressures they sight, from increased collaboration and knowledge sharing across all groups, including investigators, ethicists, and participants.

## Discussion

Researchers often navigate competing pressures, which are at times directly at odds with one another. These conflicts of interest include structural pressures, such as the need to continue to publish scientifically significant findings to secure competitive grant funding, while also adequately supporting trainees, contending with pressures to innovate, protect patients, or share data. Simultaneously, researchers grapple with interpersonal conflicts of interest that include navigating dual role relationships, prioritizing patient care, and navigating boundaries between research and care that are not always clearly delineated. Engagement and collaborations with each other, ethicists, and participants might help mitigate these pressures, but current efforts are insufficient, lacking both concrete solutions for persistent ethical challenges and a culture of support for investigators to engage with each other.

### Navigating Conflicting Pressures

These interviews suggest, first, that the ethical space encompassing conflicts of interest is much larger and more complex than often described. The literature, as well as many existing ethical conduct of research trainings, often focus on financial conflicts of interest, or the conflict of dual roles on recruitment and consent. Second, these results suggest that despite the guidelines and regulations that now exist for the ethical conduct of invasive research on humans, researchers are still grappling with best practices and how to proceed. (This has recently been found to be the case for AI ethics, as well [53].) This may suggest that investigators don’t know where to access guidelines, that they are not sure how to apply them, that guidelines conflict, or that they do not exist for the situations investigators face. Third, interviews suggest a frustration with the state of neuroethics, noting desires for both more open dialogue amongst researchers and inclusion in ethical discussion, as well as additional concrete solutions that they could use.

These narratives could be taken to suggest that neuroethics simply needs to produce more guidelines targeted at specific experiences. But we are skeptical that this approach would be sufficient for navigating current and emerging issues. For one, the pace of neuroscientific innovation may render current guidelines obsolete or extensionally inadequate, leaving investigators who are exploring the edge of scientific progress unsupported. Indeed, many of these studies are first in human studies, shaped by funding calls promoting innovation and novelty. But more fundamentally, guidelines may not be the only kinds of tools needed to help researchers navigate the practical and complex issues they have described. Recall that the most prominent ethical issues in these interviews take the form of conflicts of interest: deep and enduring tensions requiring recognizing, weighing, and responding to multiple pressures. These tensions, we suggest, are not always best navigated by guidelines, which can be general and abstract, may miss certain types of cases, or might conflict with one another. Even when guidelines are action-guiding, they do not always determine a best course of action in a particular case, and investigators need to be able to act according to good judgment.

Recall the first quote from P13 in Table 1:

We have to get grants or we lose our jobs… I think there’s certainly a conflict there, in the sense that we need the data in furtherance of our own careers. Obviously, if you are also the person treating the patient, there’s a next level conflict there… Just speaking hypothetically, if you’re a surgeon and you’re submitting a grant, and you need data, and this is your patient, and the patient knows you’re the surgeon, right? There are some things you can’t be blinded to. And so whenever you can’t be blinded to something, I think you need to be aware of it. I don’t think it necessarily disqualifies the task, but certainly something to think about from a process standpoint. (P13)

There are no rules specific enough to determine the correct, decisive course of action for this case. That is arguably one of the benefits of rules: they are meant to apply across multiple similar, but not exactly the same, types of situations. They sacrifice micro-specificity for applicability, and in this way, are essential for regulating large bodies of actions equally.

What P13 is describing are multifaceted conflicts, created not just by their own personal interests, but by existing norms, guidelines, and conceptions of societal benefit, coupled with novel research contexts, human participants, and considerable uncertainty about the downstream benefits of this research. Exactly what is right to do also depends on specific features of their situation. For example, what is the relationship between this researcher and their participants? What is the extent of the pressure this investigator is under to secure grants? The investigator must weigh all of these considerations and decide what to do on a very granular level: they don’t just need to decide whether they should consent their own patients and get the data they need, but when and where to do it, what exact words to use in the consent form and in conversation, and how to remain aware of these pressures throughout the duration of the study.

The data in these interviews suggest that despite the numerous guidelines and regulations offered to investigators, conflicts of interest persist, and researchers continue to grapple with them. To this end, we suggest that neuroethics might expand its efforts beyond producing guidelines to identifying and promoting the development of certain skills that would equip researchers to better navigate ethical dilemmas as they emerge. One way to make this shift, we suggest, is to embrace the methods of virtue ethics, a tradition that emphasizes the role of developing practical reasoning skills and moral character. In conjunction with developing guidelines and regulations, a virtue ethical approach to neuroethics would empower researchers to resolve issues in real time by developing what we will call *neuroethical competencies:* the skills, communicative capacities, and perceptive sensitivities necessary to navigate ethical questions and conflicts of interest in practice.

In what follows, we describe some core features of virtue ethics and how they might be applied to the ethics of cutting-edge neuroscience research to promote the development of neuroethical competencies and subsequently, support ethical action. We begin the project of determining and building such neuroethical competencies by suggesting two starting points: (1) developing targeted skills-based neuroethics education, and (2) promoting increased opportunities for engagement amongst investigators, ethicists, participants, and other stakeholders.

### Lessons from Virtue Ethics

At present, a significant portion of neuroethical research is dominated by proposing practices that are justified by consequentialism and deontology—that is, by outcome-oriented or duty-based considerations.[54] The ethics of brain research with human participants has arguably borrowed from both approaches.

For example, the ethical permissibility of invasive brain research with humans (the form of research conducted by the investigators in this study) relies in part on the consequentialist logic that the potential risks to participants are morally justified because they are outweighed by the potential benefits of these studies to society. Discussions of informed consent, confidentiality, and post-trial obligations in brain research with humans often have deontological rationales (e.g., adherence to duties, professional codes of conduct, and respect for the personhood and autonomy of participants). Further sets of duty-oriented guidelines related to brain research with humans can be found in the Federal Commons Rule, the BRAIN Road Map, FDA guidelines, and the Institute of Electrical and Electronics Engineers Framework [55, 56]. But a third approach, virtue ethics, which is prominent in philosophical bioethics, is largely absent in mainstream neuroethics research and practice.

Over the past few decades, virtue ethics has had a renaissance in clinical spheres: in a recent survey of bioethicists and clinical ethics, virtue ethics had the highest number of self-assessed endorsers, as compared to other popular normative theories [57]. It animates portions of bioethics [58, 59], medical and clinical ethics [60–63], care ethics and nursing ethics [64–67], and it is embraced in medical education (e.g., through attention to professional identity formation, compassion, and empathy as professional ideals) [68–70]. Neuroethics, and in particular, the ethics of rapidly evolving invasive brain research, might benefit from a similar endorsement.

The ability to identify and weigh the morally relevant features of a particular, unique situation is a core feature of virtue ethics [62, 71, 72]. Virtue ethics centers moral education, character growth, and the development of sensitivities that enable individuals to act well, or *practically reason*, across contexts, particularly where the correct action is unclear or where there are conflicting duties or guidelines in place [73–75]. We often develop the virtues that enable practical reason via moral education, exposure to cases, personal experience and reflection, engagement with and reflection upon the experiences of others, habituation, and practice. Virtue ethics directs one neither to outcomes nor duties, exclusively: it asks individuals to consider both, as well as any other relevant moral considerations [76–79].

This does not imply that rules and guidelines are useless or that researchers should not try to bring about aggregate benefit, but rather that neither is sufficient on its own for addressing the kinds of concrete ethical decisions investigators must make. Integrating a virtue ethical approach would support developing the skills and competencies that might empower investigators to practically reason about what to do when faced with complex conflicts of interest, either in conjunction with existing rules or in the absence of them.

A general note about the distinction between guidelines and what we are calling neuroethical competencies is warranted. While both can be action-guiding, creating guidelines and deploying them are forms of applied ethics. They are important: in many cases, researchers need to be directed towards what to do, and many situations require across-the-board compliance to ensure justice, fairness, and safety. Competencies, as we conceive of them, are more akin to internal character features, habits of ethical thought or consideration, or *virtues* that dispose moral agents to behave in certain ways by shaping their moral perceptions, thought processes, and communicative behavior. Developing and employing them is more akin to professional identity development than it is to applied ethics. Competencies play a role one’s ability to use guidelines when applicable and available, and to develop them in the first place. They also play a role in being able to discern where guidelines fall short. We are suggesting that identifying and developing certain neuroethical competencies—beyond the capacity to apply and follow guidelines—would help researchers meet the challenges they describe. Such an approach would also speak directly to the narratives in this study.

### Developing Neuroethical Competencies

When interviewees were asked about their interactions with neuroethics, they offered two considerations (Table 3). First, neuroethics should provide practical solutions to concrete problems, and second, neuroethics deliberation and discussion is something that researchers either benefited from in the past or desired to participate in moving forward. Perhaps unsurprisingly, we think these desiderata would be well served by things like ethics education, decision aids, increased opportunities for diversified experiences with other researchers and participants, and increased mechanisms for engagement and collaboration. Below, we suggest two steps that the field of invasive brain research could take to move towards filling these gaps. The first is developing targeted neuroethics education that prioritizes increased opportunities for practical experience, and the second is prioritizing opportunities for equitable engagement with ethicists and participants.

#### Expanding Neuroethics Education to Target Opportunities for Practical Experience

While there exist targeted neuroethics funding calls and research programs, we currently know of very few targeted neuroethics curricula, modules, or training activities. But the same logic that supports the need for the former supports the need for the later. This might be achieved by simply developing specialized training modules which highlight and expose researchers to unique neuroethical challenges and their prevailing solutions; but it also might require embracing other educational methods that have been useful in medical ethics education and which build practical skills. The ethics education of medical students, residents, and professionals often happens in simulated sessions with patient actors, morbidity and mortality rounds, Schwartz rounds, communication training, team-based learning sessions, narrative medicine writing and other medical humanities activities, and focus groups or panel discussions with patients [80–82]. Targeted neuroethics education could also expand to expose researchers to the methods of community-based participatory research, user-centered design, and other models of engagement, which are becoming increasingly recognized as requirements for ethical neurotechnology development.

These approaches, which involve experiential learning, role play, and critical discussion, often aim at the integration of ethics training with medical training, with the goal of promoting ethical physicians, as opposed to training physicians who can follow ethics principles [83, 84]. Neuroethics might focus on developing these educational activities, with an emphasis on providing opportunities to expand practical experiences, make concrete decisions in interprofessional teams, and critically reflect on those decisions as a field.

#### Prioritizing Bidirectional Engagement with Researchers, Ethicists, Participants, and Other Parties

Arguably, the parties invested in cutting edge brain research with humans are differently situated to see, appreciate, and critically assess various ethical dimensions of this research. Neuroethicists have professional expertise to think through complex ethical problems. Past and current research participants have unique knowledge that spans both what it’s like to participate in invasive research and also what it’s like to navigate the world with certain disabilities. Carepartners have knowledge about what it’s like to take care of participants and navigate complex health and social structures. Investigators have unique expertise regarding the scientific and practical details the research. No group, on their own and in isolation from the others, has the knowledge required to best navigate the complex conflicts of interest researchers describe. The frustration of some investigators with neuroethicists for not providing concrete solutions is thus perhaps misplaced: ethicists cannot do this on their own.

A better approach views ethicists, participants, and other parties as equal partners on an interprofessional team, who can learn from one another’s expertise, and together develop the tools and skills needed to address ethical issues as they arise. Facilitating opportunities for ethics conversations and team building between investigators, ethicists, and research participants might be one way to promote the skills required to address complex ethical questions, including conflicts of interest. Peer-to-peer engagement between investigators would also facilitate these skills, exposing researchers to the experiences of other teams, learning from real cases, and trouble-shooting solutions together. The ability to collaborate and synthesize multiple disparate kinds of information (clinical, scientific, experiential, ethical, political) might be a primary neuroethical competency that would better enable concrete solution-finding as well as successful inclusion in neuroethics deliberations.

These suggestions are not radical. Their underlying motivations might also be said to support the practice of embedding ethicists into neuroscience research teams. One of the benefits of this practice is that it serves as a kind of experiential education: it helps ethicists learn about the neuroscience they are attempting to guide, it serves as method for developing neuroethicists’ scientific sensitivities, and it helps them perceive the aspects of neuroscience research that are most relevant to ethical consideration. But while this approach requires ethicists to learn more about science, it doesn’t require neuroscientists to learn more about ethics. It also does not equitably include participants’ views and experiences, nor does is encourage critical reflection on how to balance scientific goals with ethical ones. Instead, we are suggesting the utility of embedding neuroscientists in real ethical deliberations with their colleagues, ethics experts, and study participants.

Taking a step back, neuroethicists and neuroethics funding might promote the efforts we have suggested by working to identify (i) what moral reasoning skills researchers need to best navigate these situations, (ii) which of these skills should be prioritized, and (iii) what tools, processes, and aids would best assist researchers in collective moral deliberation at the forefront of scientific discovery.

We are not arguing that researchers need to be neuroethicists, in the professional sense of the term. But we do think that to address the critical problems they identify, looking to the traditional approaches to and outputs of neuroethics scholarship won’t suffice. Other neuroethicists have advanced a related critique of neuroethics as proliferating rules and normative guidelines, with little attention “to *who* will read such guidelines, *how* they will see them, and *if* they can be actually implemented in practice”[85]. As the critique continues, “the situation has been one in which more and more recommendations are being churned out on similar topics—yet instead of being actualized, they quietly languish behind journal paywalls.” While not the same critique as the one we have been advancing, this sentiment certainly corroborates the idea that something is missing. What the interviews in this study suggest is that one of the missing pieces is an attention to building skills which would help researchers practically reason about what to do, with or without guidelines.

## Conclusion

The interviews presented here offer a window into the ethical issues and complex conflicts of interest that researchers conducting cutting edge brain research continue to face, despite emerging ethical rules, guidelines, and regulations. We have suggested one way to do better: to turn from ethical rules to ethical skills, reflecting the virtue ethical ideal that neuroethics is as much about the moral competencies and character of researchers as it as about the outcomes of their studies.
